# Tripartite parasitic and symbiotic interactions as a possible mechanism of horizontal gene transfer

**DOI:** 10.1002/ece3.7550

**Published:** 2021-04-06

**Authors:** Chaoyang Zhao, Shaoming Miao, Yanfang Yin, Yanjuan Zhu, Paul Nabity, Raman Bansal, Chenxi Liu

**Affiliations:** ^1^ Department of Botany and Plant Sciences University of California Riverside Riverside CA USA; ^2^ Sino‐American Biological Control Laboratory Institute of Plant Protection Chinese Academy of Agricultural Sciences Beijing China; ^3^ USDA‐ARS San Joaquin Valley Agricultural Sciences Center Parlier CA USA

**Keywords:** effector, lateral gene transfer, phospholipase C, plant‐insect interaction, salivary gland

## Abstract

Herbivory is a highly sophisticated feeding behavior that requires abilities of plant defense suppression, phytochemical detoxification, and plant macromolecule digestion. For plant‐sucking insects, salivary glands (SGs) play important roles in herbivory by secreting and injecting proteins into plant tissues to facilitate feeding. Little is known on how insects evolved secretory SG proteins for such specialized functions. Here, we investigated the composition and evolution of secretory SG proteins in the brown marmorated stink bug (*Halyomorpha halys*) and identified a group of secretory SG phospholipase C (PLC) genes with highest sequence similarity to the bacterial homologs. Further analyses demonstrated that they were most closely related to *PLCs* of *Xenorhabdus*, a genus of Gammaproteobacteria living in symbiosis with insect‐parasitizing nematodes. These suggested that *H. halys* might acquire these *PLCs* from *Xenorhabdus* through the mechanism of horizontal gene transfer (HGT), likely mediated by a nematode during its parasitizing an insect host. We also showed that the original HGT event was followed by gene duplication and expansion, leading to functional diversification of the bacterial‐origin PLC genes in *H. halys*. Thus, this study suggested that an herbivore might enhance adaptation through gaining genes from an endosymbiont of its parasite in the tripartite parasitic and symbiotic interactions.

## Graphical abstract

Halyomorpha halys acquired phospholipase C (PLC) genes from Xenorhabdus through the mechanism of horizontal gene transfer (HGT), likely mediated by a nematode during its parasitizing an insect host. This study revealed that an herbivore might enhance adaptation through gaining genes from an endosymbiont of its parasite in the tripartite parasitic and symbiotic interactions.

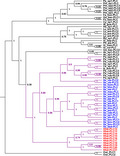

## 1 INTRODUCTION

Herbivores represent one of the most successful groups of animals on Earth that obtain energy and nutrients through consuming plant materials. Compared with other diets (*e.g*., animal source foods) plants provide greater biomass for consumption yet also evolved complex and sophisticated defense mechanisms to deter herbivores and protect nutrients behind physical barriers such as cell walls (Mitchell et al., 2016; Sarkar et al., 2009; War et al., 2012). Hence, to become a successful herbivore, one must be able to overcome plant defense and break down or bypass cell walls to release the nutrients within.

For herbivorous insects that evolved stylet mouthparts to punch plant tissues and suck plant sap, for example many hemipteran species, salivary glands (SGs) play important roles in colonization and digestion, including the suppression of plant defense and the extra‐oral digestion of plant tissue contents during feeding (Zhu et al., 2016). Connected with the mouthparts via the salivary ducts, insect SGs synthesize and secrete proteins as saliva constituents, which are then injected into plant tissues through mouthparts to prepare the optimal feeding environments (Baptist 1941; Miles, 1968; Ribeiro, 1995). In addition to directly detoxifying the secondary phytochemicals and breaking down the plant macromolecules, insect salivary proteins may act as host‐manipulating molecules, called effectors, to reprogram the plant innate immunity signaling pathways and stimulate the accumulation of plant nutrients at the feeding sites, for example, by inducing the formation of plant galls, abnormal growth of plant tissues favorable to insect feeding (van Bel & Will, 2016; Sharma et al., 2014; Xu et al., 2019; Zhao et al., 2015). Thus, secretory SG proteins are critical players of insect–plant interactions that largely determine the host range of an insect (Barrett & Heil, 2012), and dissecting the transcriptome of insect SGs provides insights into the molecular mechanisms of insect herbivory and the evolutionary history of host‐interacting proteins in insects.

Insect secretory SG proteins, especially the host‐interacting proteins (*e.g*., effectors), remain largely unexplored regarding their evolutionary origins. In plant pathogens, new effectors can emerge through gene duplication, the acquisition of a secretion function, or the mechanism of horizontal gene transfer (HGT) from a different species (Sánchez‐Vallet et al., 2018). While duplication of an existing effector gene and the acquisition of a secretion function accompanied with SG expression were suggested to contribute to the evolvement of new effectors in insects (Drurey et al., 2019; Zhao et al., 2015, 2016), HGT has yet been shown a mechanism for new insect effector emergence. By contrast, studies demonstrated that HGT‐associated gene acquirement is not rare in insects and has often conferred new functions to the gene recipients (Wybouw et al., 2016). Hence, insects could also evolve new effector‐including SG proteins through HGT to enhance adaptation.

In this study, we sought to identify and characterize the secretory SG proteins in the brown marmorated stink bug (*Halyomorpha halys*), a polyphagous hemipteran species that has dramatically increased its populations globally with high versatility to feed on a wide array of plants (Valentin et al., 2017). Although its SG transcriptomes were previously sequenced (Liu & Bonning, 2019), we performed re‐sequencing and transcriptome analysis for the following reasons. First, we attempted to generate a high‐coverage dataset for the discovery of all possible important genes by sequencing multiple replicates of insect tissues. Second, the *H. halys* genome was sequenced recently (Sparks et al., 2020), which allowed the improvement of the transcriptome annotation (Freedman et al., 2020). And third, the focus of this study was on the secretory proteins, especially those associated with HGT, which was not reported in the previous analysis (Liu & Bonning, 2019). We predicted hundreds of secretory SG proteins, many of which formed sequence similarity‐based clusters and may function as digestive enzymes or plant‐manipulating effectors. Interestingly, several proteins showed highest sequence similarity to the microbial homologs, suggesting that the insect acquired these genes through HGT. Further analyses indicated that a group of horizontally transferred genes that encode phospholipase C (PLC)‐like phosphodiesterase, TIM beta/alpha‐barrel domain‐containing protein, called PLC hereafter, were closest to homologs of *Xenorhabdus*, a bacterial genus whose members are obligate symbionts of entomopathogenic nematodes (Sajnaga & Kazimierczak, 2020; Thomas & Poinar Jr, 1979). Thus, this study revealed a possible novel HGT mechanism likely associated with the tripartite bacterium–insect–nematode interaction that is co‐opted by herbivores to facilitate plant feeding.

## 2 MATERIALS AND METHODS

### 2.1 Insect collection and rearing

*Halyomorpha halys* adults were originally collected from the field in Beijing, China. To maintain the population in the laboratory, the bugs were reared in cages covered with a fine nylon mesh net in a growth chamber at 27°C with a 16:8 light dark cycle and were fed with water and cowpea (*Vigna unguiculata*) leaves.

### 2.2 Salivary gland RNA sequencing and transcriptome assembly

Salivary glands, including both the principle and accessory glands, were collected from *Halyomorpha halys* adults for total RNA extraction. mRNA molecules were enriched using the oligo‐dT beads and libraries were constructed for RNA sequencing on the Illumina HiSeq 2,500 platform to generate 100‐bp paired‐end reads. Raw reads were cleaned by removing the adapters and low‐quality sequences using Seqtk (https://github.com/lh3/seqtk) to generate a de novo RNA‐seq assembly with the Trinity program by its default parameters (Grabherr et al., 2011). The assembled transcripts were lastly collapsed into UniGenes by selecting the longest transcript per transcriptome locus, and the completeness was assessed using BUSCO (v3.0.2) (Simão et al., 2015) against the insect database (insect_odb9).

### 2.3 Prediction and annotation of salivary gland secretory proteins

*Halyomorpha halys* salivary gland UniGenes were translated into protein sequences (≥ 100‐aa) with a standalone Perl script (Min et al., 2005). To predict secretory salivary gland proteins, the translated sequences were first screened for the complete N‐termini featured by methionine as the start amino acid, and the selected sequences were subsequently predicted for the presence of N‐terminal secretory signal peptides using SignalP (v4.1) but not containing transmembrane domains using TMHMM (v2.0) (Carolan et al., 2011; Zhao et al., 2019).

To annotate these putative secretory SG proteins, their corresponding gene models in the *H*. *halys* genome, if available, were identified through BLASTP searches against the *H. halys* genome protein database (Sparks et al., 2020) based on a threshold of >95% sequence identity and <1e−5 E‐value. The hit proteins were filtered by the same criteria, that is, containing N‐terminal signal peptides but no transmembrane domains and were subsequently collapsed into genome loci by choosing only one protein (the longest protein sequence, in case of >1 isoforms detected) as the representative of each gene.

For functional annotation, these secretory salivary gland proteins were first used to search (E‐value <1e−3) against the NCBI non‐redundant (NR) database (updated until 2019/03/22), and the gene ontology (GO), enzyme code, and InterPro analyses were run on Blast2GO (Götz et al., 2008). Lastly, these proteins were grouped based on their sequence similarity using OrthoMCL (v2.0.9) with E‐value ≤1e‐10 and inflation rate =1.2.

### 2.4 Identification of PLC genes from the *H. halys* genome

To identify all possible PLC genes encoded by the *H. halys* genome, 25,026 genome‐wide protein sequences were screened for the PLC‐like phosphodiesterase, TIM beta/alpha‐barrel domain superfamily (IPR017946) using InterProScan. The hit proteins were mapped to the genome loci. Only one protein sequence (the longest one) per gene locus was retrieved for phylogenetic analysis.

### 2.5 Phylogenetic analysis

We performed two phylogenetic analyses on the obtained PLC proteins: the general PLC phylogenetic analysis and the HGT‐related PLC phylogenetic analysis. For the former, PLC sequences were selected from a variety of organisms including plant (*Arabidoposis thaliana*), mammal (*Mus musculus*), insects that included *Drosophila melanogaster* and several orders of species, spider (*Parasteatoda tepidariorum*), mite (*Tetranychus urticae*), fungus, and bacterium species that belong to a number of phyla, and two *D. melanogaster* PLA proteins were used as outgroups (Table S1). For the latter, we used a bacterium‐derived Hhal‐PLC protein sequence (XP_014286808.1) to search against the NCBI database and all 55 Gammaproteobacteria sequence hits with E‐value <1e−10 were pooled along with the 10 horizontally transferred Hhal‐PLCs to construct a phylogenetic tree which set Bacteroidetes PLCs as outgroups (Table S1).

To build the phylogenetic trees, protein sequences were aligned using MAFFT (v7.427), and the poorly aligned regions were trimmed using TRIMAL (v1.4.1) based on a gap threshold of 0.25. The best‐fit models of protein evolution, which were LG+G for the general PLC phylogenetic analysis and WAG+G + F for the HGT‐related phylogenetic analysis, were determined using ProtTest (v3.4.2) according to Bayesian information criteria (Darriba et al., 2011). Phylogenetic analyses were conducted using two runs with 4 chains per run in MrBayes (v3.2.6) until the standard deviation of split frequencies between runs dropped below 0.05. The first 25% of generations were discarded, and the remaining generations were used to construct a 50% majority‐rule consensus tree.

### 2.6 Quantitative reverse transcription PCR

Total RNA was extracted from the head (excluding salivary gland), salivary gland, midgut, and muscle of *H. halys* adults and was quantified using a NanoDrop 2000 spectrophotometer (Thermo Scientific, USA). First‐strand cDNA was synthesized using HiScript^®^ II Q RT SuperMix with gDNA wipe and the Oligo(dT)23VN primer (Vazyme Biotech, Nanjing, China). Quantitative reverse transcription PCR (qRT‐PCR) was carried out on LightCycler^®^ 480 II (ROCHE, Basel, Switzerland) with a reaction volume of 10 μl mixture containing 1 μl of cDNA, 5 μl of 2×QuantiFast^®^ SYBR^®^ Green PCR Master Mix (Qiagen, Germany), 0.2 μl of forward primer, 0.2 μl of reverse primer and 3.6 μl of nuclease‐free water. The sequences of PCR primers are listed in Table S2. The PCR involved a 95℃ step for 5 min followed by 40 cycles at 95℃ for 10 s, 60℃ for 30 s. Raw data from qRT‐PCR were obtained as cycle threshold (Ct) values determined by single threshold mode. A standard curve was obtained for each set of primers to ensure 90%–100% efficiency. All reactions were run in triplicate with four independent biological replicates. The expression levels of mRNAs were normalized against *H. halys* 18S rRNA (Mogilicherla et al., 2018) and were calculated using the 2^‐ΔΔCt^ method (Livak & Schmittgen, 2001). Data on qRT‐PCR were analyzed using one‐way ANOVA (SPSS, Chicago, IL, USA) with Dunnet's Multiple Comparisons test.

## 3 RESULTS

### 3.1 Transcriptome assembly and secretory salivary gland protein identification

We collected the salivary gland tissue from adult *H. halys* and sequenced the transcriptome using the Illumina HiSeq 2,500 platform. Four replicates of paired‐end RNA‐seq reads were pooled, yielding a total of 190.8 million raw reads, which, after trimming, generated 186.6 million clean reads. Using these trimmed reads, we assembled a de novo transcriptome consisting of 67,108 transcripts, from which the longest transcript of each transcription locus was extracted to generate 53,618 UniGenes with a mean length of 736‐bp. To assess the completeness of this assembly, we performed BUSCO analysis, revealing the presence of 94.9% BUSCO genes, including 91.0% complete genes and 3.9% fragmented genes, with only 5.1% missing in this transcriptome assembly.

To annotate the salivary gland transcriptome, 53,618 UniGenes were translated into 11,026 proteins with minimum length of 100‐aa, out of which 9,871 sequences containing putatively complete N‐termini were selected for the prediction of secretory characteristics. 691 were predicted to be secretory owing to the presence of N‐terminal signal peptide and absence of transmembrane domain. We further identified 523 of their corresponding genome‐derived gene models (Sparks et al., 2020). After filtering with the secretory feature (presence of signal peptide and absence of transmembrane domain) and selecting only a single gene model per gene locus, we finally obtained 430 secretory proteins, each representing a single *H. halys* gene.

### 3.2 Functional annotation of secretory salivary gland proteins

**FIGURE 1:**
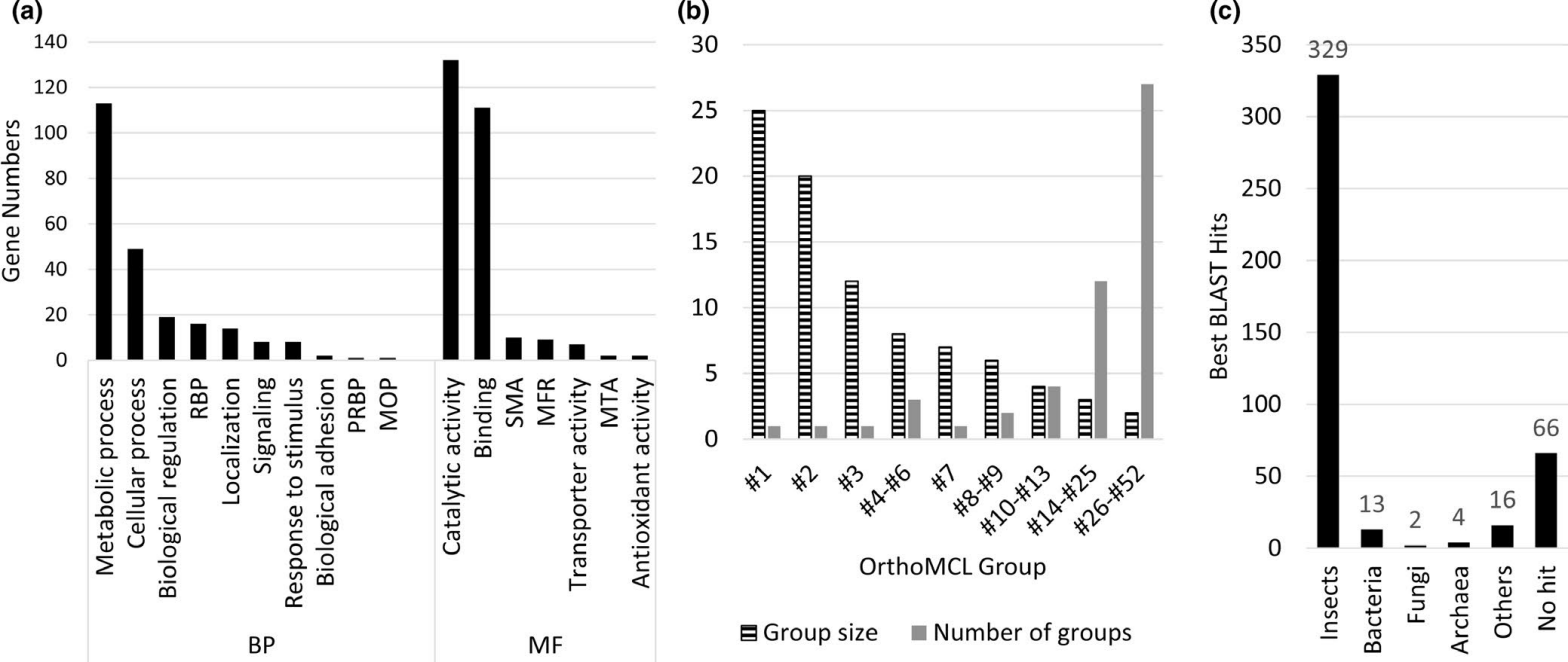
Annotation of predicted secretory salivary gland proteins in *H. halys*. (a) Gene ontology terms assigned to protein sequences involved in biological process (BP) and molecular function (MF). In the BP terms, RBP = regulation of biological process, MOP = multicellular organismal process, PRBP = positive regulation of biological process. In the MF terms, SMA = structural molecular activity, MFR = molecular function regulator, MTA = molecular transducer activity. (b) Protein sequences classified into OrthoMCL groups based on sequence similarity. Stripped bars (group size) indicate the number of gene members included in a group, and grey bars (number of groups) indicate the number of OrthoMCL groups having a same group size. (c) Species distribution of best BLAST hits in the NCBI NR database with *H. halys* excluded for the predicted secretory salivary gland proteins

To predict the functions of the secretory salivary gland proteins, we performed GO analysis based on Biological Process and Molecular Function. GO terms could be assigned to 260 of the 430 proteins (Figure 1a; Table S3). In the category of Biological Process, 113 and 49 GO terms were assigned to Metabolic Process and Cellular Process, respectively, accounting for the most prevalent GO terms. In the category of Molecular Function, the most abundant GO terms are Catalytic Activity (132) and Binding (111) (Figure 1a).

Sequence similarity‐based clustering assigned 206 secretory salivary gland proteins into 52 OrthoMCL groups, leaving the remaining 224 proteins to be singletons (Table S3). Of the clustered proteins, 25, 20, and 12 formed the three largest groups (#1, #2, and #3), respectively, and a large number of proteins formed multiple small‐sized groups which included 2–4 members each (Figure 1b). Interestingly, the largest group (Group #1) of proteins are serine‐type endopeptidase (IPR001314), a type of enzyme that cleaves peptide bonds. The second largest group, Group #2, contains ankyrin repeat motifs (IPR002110), a type of protein involved in protein–protein interactions (Table S3).

By searching the homologs in the NCBI NR database without self‐hitting, that is, excluding *H. halys* proteins in the database, we found that although the majority (329 or 76.5%) of secretory salivary gland proteins had the best hits from insect species as expected, a small number of proteins surprisingly had those of microorganisms, including bacteria, fungi, and archaea, as the best hits (Figure 1c). The 13 proteins that were most closely related to bacterial homologs included five ankyrin proteins (Group #2), four phosphoric diester hydrolases, specifically PLCs (Group #12), three beta‐mannosidase (Group #24), and one leucine‐rich repeat‐containing protein (singleton) (Table S3). Interestingly, Group #2 comprises ankyrin proteins sharing highest sequence similarity, based on the BLAST search, to the homologs of organisms derived from a variety of taxa, including insect, Archaea, bacterium, and vertebrate (Table S3), suggesting that *H. halys* might have gained genes through HGT from different sources.

### 3.3 Evolution of PLC genes in *H. halys*

**FIGURE 2:**
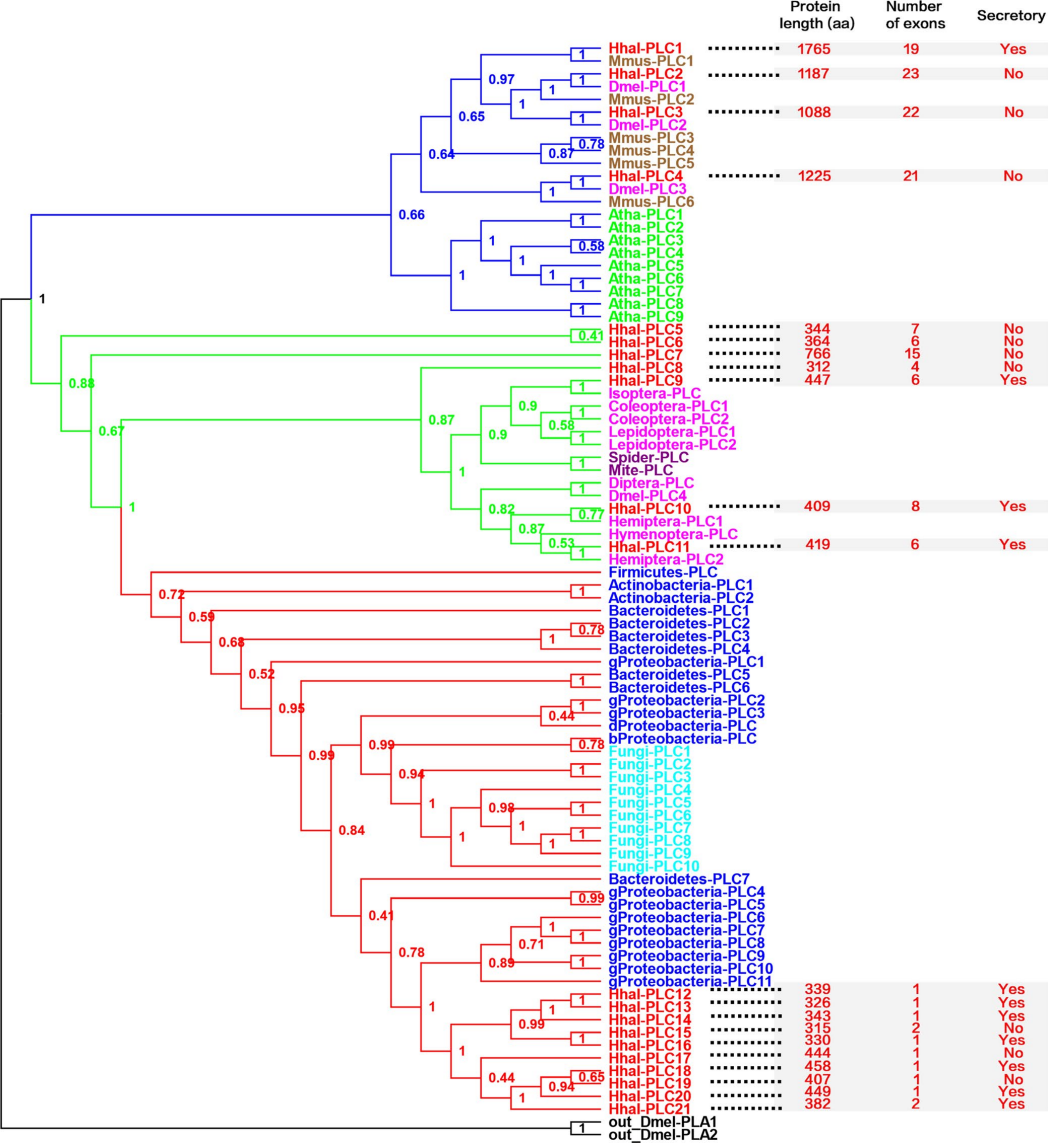
Phylogeny of PLC proteins derived from *H. halys* and a variety of kingdoms of organisms. The eukaryote, arthropod, and bacteria PLC clades are in blue, green, and red, respectively. Clade posterior probabilities are indicated at nodes. Taxa are color coded per organism lineage with the outgroup PLA proteins in black, *H. halyes* (Hhal) in red, *Mus musculus* (Mmus) in brown, *Arabidopsis thaliana* (Atha) in green, *Drosophila melanogaster* (Dmel) and other non‐Hhal insect species (shown in Order names) in pink, arachnids in purple, bacteria in blue, and fungi in cyan. Bacteria species are shown in their phylum names except Proteobacteria which are shown in class names, that is, bProteobacteria = Betaproteobacteria, dProteobacteria = Deltaproteobacteria, and gProteobacteria = Gammaproteobacteria. Detailed taxa information is in Table S1

Given that a number of *H. halys* secretory SG proteins are most similar to the homologs of microorganisms, it is likely that the insect genes encoding these proteins were acquired from microorganisms through HGT. To test this, we selected the proteins in OrthoMCL Group #12 for a comprehensive phylogenetic analysis. InterPro analysis indicated that these groups of proteins are members of phospholipase C (PLC), TIM beta/alpha‐barrel domain‐containing protein (IPR017946) (Table S3). Through InterProScan search, we identified 21 such PLC proteins including four that were predicted as secretory salivary gland proteins from the *H. halys* genome (Figure 2).

To investigate how these 21 *H. halys* PLC genes evolved, we constructed a phylogenetic tree using the PLC proteins sequences derived from a variety of organisms including bacteria, fungi, plant, mammal, insects, and arachnids (Figure 2). Interestingly, 21 *H. halys* PLCs were clustered in three clades. The first clade, named the Eukaryote Clade, comprised four Hhal‐PLCs (1–4) and PLCs of mammal, insect, and plant. The second clade, named the Arthropod Clade, comprised seven Hhal‐PLCs (5–11) and PLCs of all arthropod species including various Orders of insect species and arachnids. The third clade, designated as the HGT Clade here, however, only comprised 10 Hhal‐PLCs (12–21) and bacterial and fungal PLCs. Noteworthily, these 10 Hhal‐PLCs were clustered together with Gammaproteobacteria PLCs with strong support (posterior probability = 1), indicating that the genes encoding these 10 Hhal‐PLCs were gained by *H. halys* from the class of Gammaproteobacteria through the mechanism of HGT (Figure 2). The 10 Hhal‐PLCs forming a unique subclade also indicated that the horizontal transfer of PLC gene from Gammaproteobacteria to *H. halys* was a single event, which was followed by gene duplication to expand this type of genes in the insect genome. Given that these Hhal‐PLC genes are typically adjacent to other insect conserved genes in the genome (Sparks et al., 2020), for example, approximately 15‐kb downstream of *Hhal‐PLC12* (LOC106678613) is the *mRNA‐lymphokine‐activated killer T‐cell‐originated protein kinase* gene (LOC106678610) whose top BLAST hits against NCBI Non‐Redundant database were all from insect species, it is unlikely that they were derived from the contaminant symbionts during the genome sequencing. To further rule out this possibility, we extracted the genomic DNA from the legs of *H. halys*, the tissue where symbiont contamination is less likely to occur, for gene‐specific amplification of the fragments from four HGT‐related Hhal‐PLC genes (Table S2). Results showed that expected sizes of DNA fragments were amplified from all four genes (Figure S1) with verified sequences by Sanger sequencing, supporting that these genes were derived from the HGT event.

Sequence analyses also suggested that the three clades of Hhal‐PLC genes evolved independently. Unlike the Eukaryote Clade of proteins that are all longer than 1000‐aa, the Arthropod and HGT Clades of proteins are typically shorter than 500‐aa except Hhal‐PLC7 (Arthropod Clade) which is 766‐aa (Figure 2). Similarly, the gene exon numbers differed in the three clades of PLCs. Compared with the Eukaryote Clade of genes that were most exon‐abundant (19–23 exons), the Arthropod Clade of genes were generally composed of 4–8 exons and only one gene (Hhal‐PLC7) was composed of 15 exons. In contrast, the HGT Clade of PLC genes mostly contained a single exon except Hhal‐PLC15 and Hhal‐PLC21 containing two exons (Figure 2). This intron‐less/intron‐poor feature of the HGT Clade of genes further implied their bacterial origin.

### 3.4 The HGT Clade of Hhal‐PLC genes originated from a bacterial genus that are in symbiosis with entomopathogenic nematodes

**FIGURE 3:**
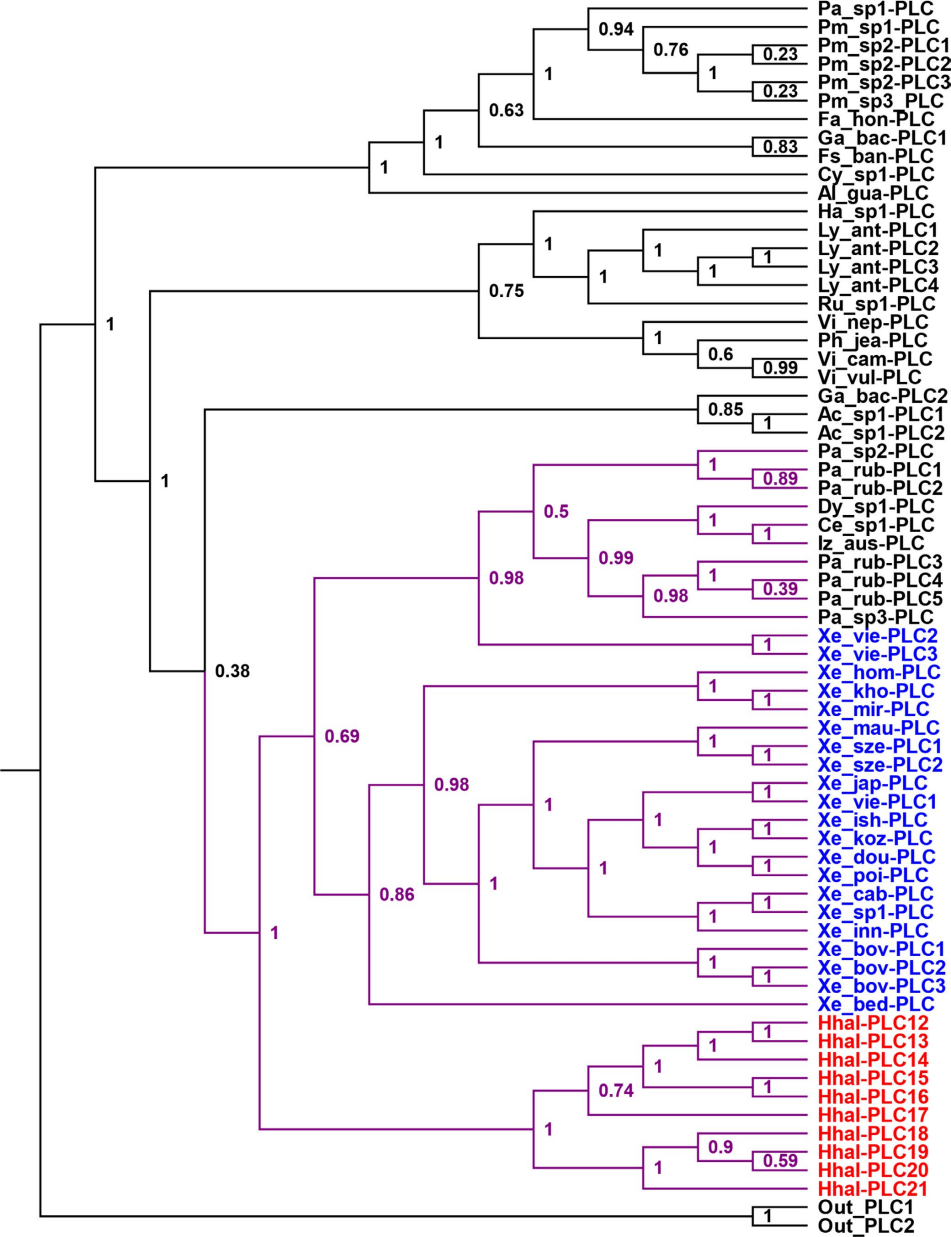
Phylogeny of the HGT‐derived *H. halys* PLC proteins and Gammaproteobacteria PLCs. The tree is rooted on two Bacteroidetes PLC proteins. Clade posterior probabilities are indicated at nodes. The clade comprising *H. halys* (Hhal) PLCs (in red) and their most closely related bacterial PLCs, particularly *Xenorhabdus* (Xe) are highlighted in purple. First two letters of the Gammaproteobacteria taxa denote the genera, and three characters following an underscore symbol denote the species. Detailed taxa information is in Table S1

Because the HGT Clade of Hhal‐PLC genes were most closely related to Gammaproteobacteria PLCs (Figure 2), we performed a high‐resolution phylogenetic analysis using all Gammaproteobacteria PLC sequences retrieved from the NCBI NR database to further trace the donor species of these HGT‐related PLC genes (Figure 3). As expected, the 10 Hhal‐PLC proteins formed a separated subclade, which, surprisingly, were clustered with another subclade consisting of sequences mainly from a bacterial genus, *Xenorhabdus*, with strong support (posterior probability = 1). Interestingly, the non‐*Xenorhabdus* PLCs in this clade formed their own subclade, showing closest phylogenetic relationship with those (Xe_vie‐PLC2 and Xe_vie‐PLC3) of *Xenorhabdus vietnamensis*, suggesting possible horizontal gene transfers occurring between these species, likely starting with a transfer from *X*. *vietnamensis* to one of these non‐*Xenorhabdus* species (Figure 3). Nevertheless, the Hhal‐PLCs were most closely related to the homologs of several *Xenorhabdus* species, indicating that the genes encoded by the insect genome and those of *Xenorhabdus* species, shared a common ancestor. Given that *Xenorhabdus* species are bacteria living in symbiosis with insect‐parasitizing nematodes (Sajnaga & Kazimierczak, 2020; Thomas & Poinar Jr, 1979), our finding suggested that the bacterium‐to‐insect transfer of the HGT Clade of PLC genes likely occurred during a *Xenorhabdus*‐harboring nematode establishing its parasitism on *H. halys*.

### 3.5 Functional diversification of the HGT Clade of Hhal‐PLC proteins

**FIGURE 4:**
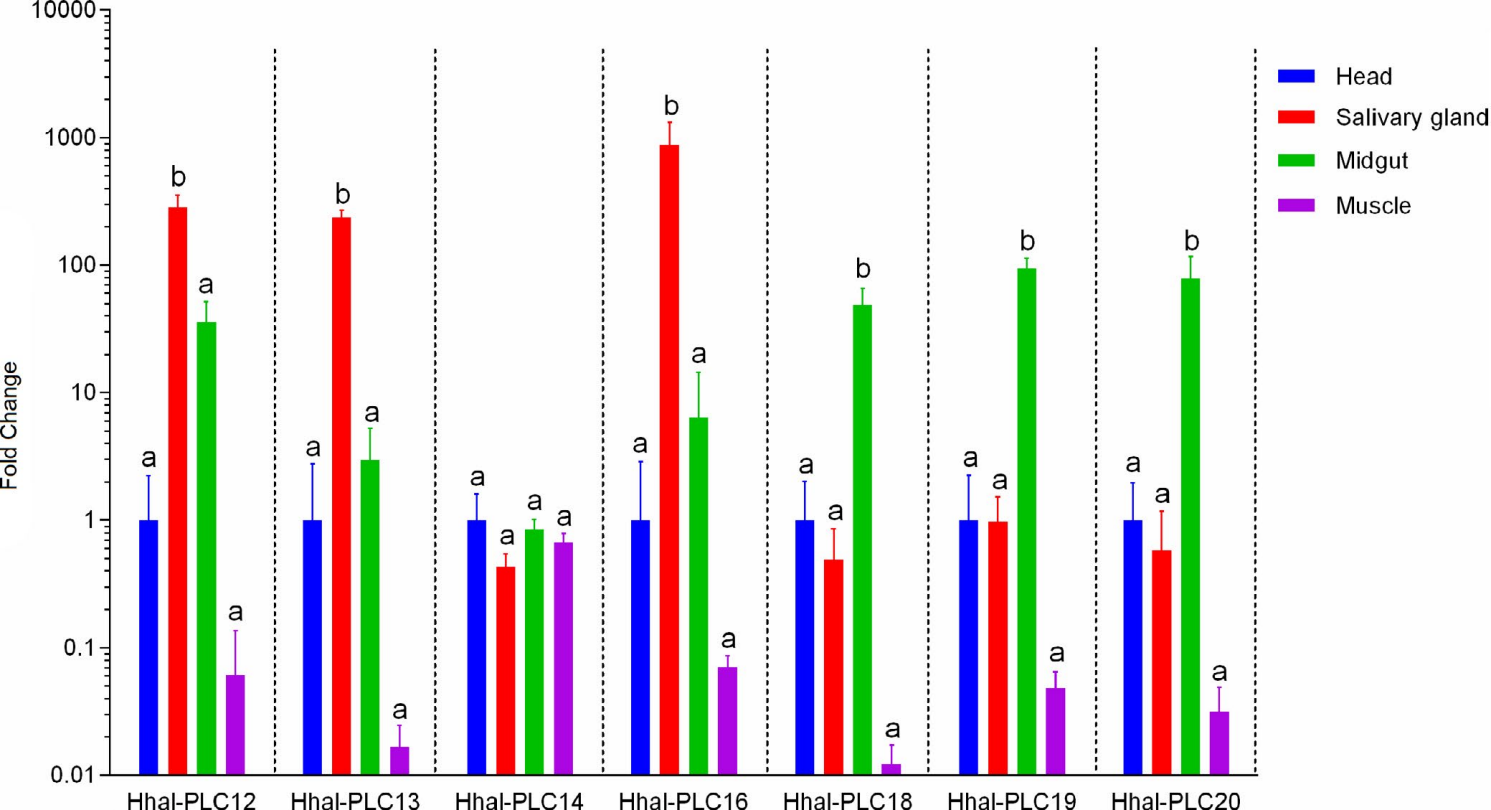
Quantitative reverse transcription PCR analysis of seven horizontally transferred PLC genes in the tissues (head, salivary gland, midgut, and muscle) of *H. halys*. Data shown are mean relative expression ±SE. Different letters on error bars indicate significant difference (*P*‐value <0.05)

To understand how the HGT Clade of Hhal‐PLCs functions in *H. halys*, we compared them with other clades of Hhal‐PLCs regarding the secretory feature, determined with the presence of N‐terminal secretory signal peptide and absence of transmembrane domain. Similar in all three clades, both secretory and non‐secretory types of PLCs were found despite that two clusters of secretory PLCs, formed by Hhal‐PLCs 12–14 and 20–21, respectively, were within the HGT Clade (Figure 2). qRT‐PCR analysis demonstrated that Hhal‐PLC12, ‐PLC13, and ‐PLC16 were expressed at significant higher level in salivary gland than other tissues tested, including head, midgut and muscle (Figure 4). In contrast, expression of Hhal‐PLC18, −19, and −20 was significantly upregulated in midgut compared with other tissues. However, Hhal‐PLC14 was similarly expressed in all these four tissues (Figure 4). The diverse expression profiles and distinct secretory features in the HGT Clade of Hhal‐PLCs indicated the genes have been evolved towards functional diversification following their horizontal gene transfer and expansion in the insect genome.

## 4 DISCUSSION

From the salivary gland secretome of *H. halys*, we identified several genes with bacterial homology, possibly gained by the insect through HGT. Despite that previous transcriptome and genome annotations allowed the detection of a number of HGT events in *H. halys*, likely with *H. halys* endosymbionts *Wolbachia* as gene donors (Ioannidis et al., 2014; Sparks et al., 2020), here we discovered a new gene transfer event with a different bacterial lineage as the donor, that is, the horizontal transfer of a PLC gene most likely from *Xenorhabdus*, a genus of bacteria mutualistically associated with a genus of entomopathogenic soil nematodes, *Steinernema* (Ferreira & Malan, 2014). Typically, a *Steinernema* nematode carries its symbiont in a specialized region of the intestine, termed the receptacle, until the nematode invades the insect hosts, penetrates their hemocoels, and releases *Xenorhabdus spp*. into the insect hemolymph. Once in the insect, the bacteria reproduce and secrete a complex of virulent factors in coordination with nematodes to suppress insect immunity and kill the insect host (Goodrich‐Blair & Clarke, 2007). The bacteria also offer essential nutrients to nematodes by degrading the insect cadaver and meanwhile produce a diversity of antimicrobial molecules to prevent the growth of antagonistic microorganisms. This specialized lifecycle of *Xenorhabdus spp*., which involves pathogenic and mutualistic interactions with different invertebrates hosts, the insects and nematodes, respectively, provides an opportunity for bacterium‐to‐insect horizontal gene transfer because the bacteria are released into an open circulatory system, potentially bringing bacterial DNA close to an insect host germ cell, and thus allowing DNA integration and transmission to the next generation if the host survives the attack.

Horizontal gene transfer events between insects and microbes have been detected in the genomes of numerous insect species (Nakabachi, 2015), and many of these HGT events associate with obligate endosymbiosis. This mutualistic association provides a stable form of physical connection and increases the probability of foreign DNA incorporation into insect genomes (Dunning Hotopp, 2011). In addition, parasitism promotes HGT between the parasites and their hosts because of similar physical connectedness (Wijayawardena et al., 2013) with a classical example of the transfer of *P*‐elements among drosophilids mediated by the ectoparasitic mite, *Proctolaelaps regalis* (Houck et al., 1991). Remarkably, studies on the interactions among the parasitoid wasp, its viral symbiont, and its lepidopteran host revealed that a wasp gene may be transferred to the host insect by the virus (Gasmi et al., 2015) and that a virus gene may also be transferred to the host by the wasp (Di Lelio et al., 2019). Occurrence of the virus‐to‐host gene transfer relies upon the symbiotic virus being injected along with the wasp egg into the body of the host (Di Lelio et al., 2019). Similarly, we present support for the first possible nematode‐mediated bacterium‐to‐insect HGT example, expanding the understanding that HGT plays an important role in promoting gene flows between reproductively isolated organisms.

A growing body of evidence showed that HGT has reshaped the genome evolution and conferred new functions to the gene recipient organisms to facilitate nutrient digestion and metabolism, detoxify host metabolites, suppress host defense, and enhance immunity (Di Lelio et al., 2019; Wybouw et al., 2016). In the present study, we demonstrated that *H. halys* acquired a bacterial PLC gene, which was later duplicated and expanded in the insect genome. These bacterial‐origin *PLCs* were encoded by the insect genome rather than derived from contaminant symbionts because they were as follows: (i) included in the insect genome sequence and adjacent to other insect conserved genes; (ii) identified by RNA Sequencing and qRT‐PCR analyses which only selected eukaryotic genes encoding poly(A) tail‐containing mRNAs; and (iii) detected from insect leg DNA with low symbiont contamination likelihood. Expression and sequence analyses revealed that these HGT Clade of genes contained both secretory and non‐secretory types with variable gene expression across tissues, indicating functional diversification of PLC genes in *H. halys* following HGT. PLC is a class of enzymes that catalyze the hydrolysis of phosphatidylinositol 4,5‐bisphophate (PIP2) into inositol 1,4,5‐trisphosphate (IP3) and diacylglycerol (DAG), both of which serve as important second messengers to regulate a variety of cellular processes (Bill & Vines, 2020). Therefore, diverse expression patterns plus intra‐ and extra‐cellular/oral forms of the horizontally acquired PLCs in *H. halys* pointed to the possibility that the non‐extra‐oral types of PLCs may function as insect's own physiological process regulators while others expressed as the extra‐oral types may be secreted to modulate the cellular signaling within the host plants. Although the role of each HGT‐associated PLC gene remains to be determined, their adoption by *H. halys* suggested that they have conferred new physiological traits beneficial to insects.

Identification of PLC genes from the *H. halys* salivary glands was not reported in the previous transcriptome analysis (Liu & Bonning, 2019), which could be that the transcriptomes generated from only a single replicate of insect tissues did not yield RNA‐seq reads in a high‐enough amount for the discovery of certain important genes. Compared with the 148.2 million raw reads and 133.8 million clean reads obtained in that study (Liu & Bonning, 2019), our RNA‐seq raw and clean read numbers, through sequencing four replicates of salivary gland samples, were increased to 190.8 millions and 186.6 millions, respectively. Similarly, our mean UniGene length (736‐bp) was greater than the mean transcript length of the previously assembled transcriptomes, which was 659‐bp for the principle gland and 654‐bp for the accessory gland (Liu & Bonning, 2019). However, both transcriptome analyses found similar overall protein compositions with the largest categories of proteins involved in Metabolic Process and Cellular Process based on the biological process GO terms, and in Catalytic Activity and Binding based on the molecular function GO terms. Consistently, our secretome analysis showed that serine‐type endopeptidases (IPR001314) and ankyrin domain (IPR002110)‐containing proteins represented the two largest OrthoMCL groups (Groups #1 and #2). Serine‐type endopeptidases have been earlier identified from the transcriptome and proteomes of *H. halys*, and were demonstrated to function as extra‐oral digestive enzymes (Liu & Bonning, 2019; Lomate & Bonning, 2016; Peiffer & Felton, 2014), which appeared to be a prevalent feeding strategy among herbivorous hemipterans (Wright, et al. 2006; Lomate & Bonning, 2016; Zhu et al., 2016). In contrast, identification of ankyrin proteins from the salivary glands/saliva of herbivorous insects was scarcely reported, except in the white‐backed planthopper (*Sogatella furcifera*) (Miao et al., 2018), and the yellow soldier fly (*Inopus flavus*) (Etebari et al., 2020), with unknown functions. Serving as linkers to mediate the protein‐protein interactions, ankyrin repeats play essential roles in a myriad of physiological processes in pro‐ and eukaryotes, including transcription and cell cycle regulation, signal transduction, and stress responses (Li et al., 2006). Notably, ankyrin proteins have been shown to be secreted by microbial pathogens to manipulate the cellular processes of their eukaryotic hosts during infection (Al‐Khodor et al., 2010; Chen et al., 2017; Pan et al., 2008). Given that the employment of the functionally homologous effectors by distantly related plant pathogens and pests, as a phenomenon of convergent evolution, to attack the conserved plant immunity signaling networks is not unusual (Jwa & Hwang, 2017; Zhao et al., 2015), it is conceivable that *H. halys* secretes the ankyrin proteins as plant‐manipulating effectors possibly to suppress plant defense when feeding.

## CONFLICT OF INTEREST

The authors declare no conflict of interest. The funders had no role in the design of the study; in the collection, analyses, or interpretation of data; in the writing of the manuscript, or in the decision to publish the results.

## AUTHOR CONTRIBUTION

**Chaoyang Zhao:** Conceptualization (equal); Data curation (equal); Writing‐original draft (equal). **Shaoming Miao:** Data curation (equal); Validation (equal). **Yanfang Yin:** Data curation (equal); Validation (equal). **Yanjuan Zhu:** Data curation (equal); Validation (equal). **Paul D. Nabity:** Data curation (equal); Writing‐review & editing (equal). **Raman Bansal:** Writing‐review & editing (equal). **Chenxi Liu:** Conceptualization (equal); Data curation (equal); Validation (equal); Writing‐original draft (equal).

## Supporting information

Figure S1

Table S1

Table S2

Table S3

## Data Availability

All the sequencing reads generated from Illumina HiSeq RNA‐Seq are available in NCBI SRA: SRX8790246‐SRX8790249. All other datasets supporting this study are included within the article and its supplementary material.
