# Supplementary figures and images for: Tripartite parasitic and symbiotic interactions as a possible mechanism of horizontal gene transfer
Source: Ecol Evol. 2021 Apr 6;11(11):7018–28. doi: 10.1002/ece3.7550 (PMC8207144; doi:10.1002/ece3.7550)

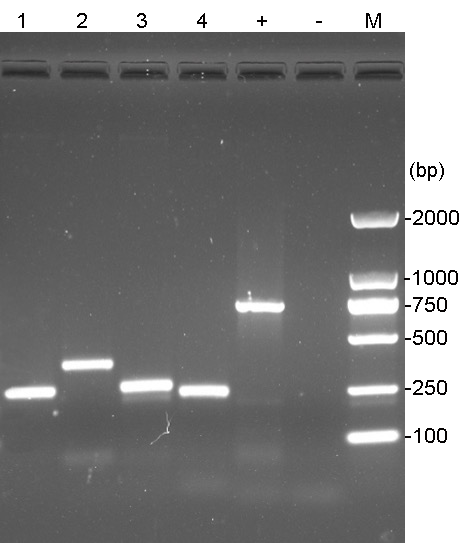

Supplement: Supplementary file 1 — Figure S1 [file ECE3-11-7018-s002.tif]
